# Stepping stones to isolation: Impacts of a changing climate on the connectivity of fragmented fish populations

**DOI:** 10.1111/eva.12613

**Published:** 2018-03-14

**Authors:** Emma F. Young, Niklas Tysklind, Michael P. Meredith, Mark de Bruyn, Mark Belchier, Eugene J. Murphy, Gary R. Carvalho

**Affiliations:** ^1^ British Antarctic Survey Cambridge UK; ^2^ School of Biological Sciences Bangor University Bangor Gwynedd UK; ^3^ School of Life and Environmental Sciences The University of Sydney Sydney NSW Australia; ^4^ Present address: INRA UMR8172 EcoFoG AgroParisTech Cirad CNRS Université des Antilles Université de Guyane Kourou France

**Keywords:** *Champsocephalus gunnari*, connectivity, individual‐based modelling, *Notothenia rossii*, ocean warming, population genetics, Scotia Sea

## Abstract

In the marine environment, understanding the biophysical mechanisms that drive variability in larval dispersal and population connectivity is essential for estimating the potential impacts of climate change on the resilience and genetic structure of populations. Species whose populations are small, isolated and discontinuous in distribution will differ fundamentally in their response and resilience to environmental stress, compared with species that are broadly distributed, abundant and frequently exchange conspecifics. Here, we use an individual‐based modelling approach, combined with a population genetics projection model, to consider the impacts of a warming climate on the population connectivity of two contrasting Antarctic fish species, *Notothenia rossii* and *Champsocephalus gunnari*. Focussing on the Scotia Sea region, sea surface temperatures are predicted to increase significantly by the end of the 21st century, resulting in reduced planktonic duration and increased egg and larval mortality. With shorter planktonic durations, the results of our study predict reduced dispersal of both species across the Scotia Sea, from Antarctic Peninsula sites to islands in the north and east, and increased dispersal among neighbouring sites, such as around the Antarctic Peninsula. Increased mortality modified the magnitude of population connectivity but had little effect on the overall patterns. Whilst the predicted changes in connectivity had little impact on the projected regional population genetic structure of *N. rossii*, which remained broadly genetically homogeneous within distances of ~1,500 km, the genetic isolation of *C. gunnari* populations in the northern Scotia Sea was predicted to increase with rising sea temperatures. Our study highlights the potential for increased isolation of island populations in a warming world, with implications for the resilience of populations and their ability to adapt to ongoing environmental change, a matter of high relevance to fisheries and ecosystem‐level management.

## 1 INTRODUCTION

Despite the plethora of studies focusing on marine connectivity (Kelley, Brown, Therkildsen, & Foote, 2016), there remains a need to better elucidate key environmental factors and life‐history stages influencing population genetic structure. Such information is vital for effective conservation management, and for predicting the potential impacts of ongoing and future environmental change, both globally and regionally. A complexity of processes is at play, resulting often in an uncoupling of dispersal potential, realized gene flow and patterns of genetic differentiation (Selkoe, Henzler, & Gaines, 2008; Selkoe et al., 2016). Whilst in some cases, variance in life‐history traits combined with general oceanographic patterns can predict connectivity (e.g., Pascual, Rives, Schunter, & Macpherson, 2017; Riginos, Douglas, Jin, Shanahan, & Treml, 2011; Young et al., 2015), interplay among oceanographic variability, mortality, and habitat availability, and potential differences in local adaptation, often obscure predictions based solely on the product of the two factors (e.g., Galarza et al., 2009; White et al., 2010).

Whilst marine connectivity is influenced by dispersal of juveniles and adults in mobile species, larval dispersal plays a primary role in determining population and species distributions, especially in highly fragmented habitats (Cowen & Sponaugle, 2009). Oceanic temperature will affect larval development, dispersal and recruitment in various ways by influencing, for example, spawning date, rates of feeding (planktotrophic) or nonfeeding (lecithotrophic) modes of development, levels of predation and the ability to reach suitable habitats and subsequent postsettlement survival (Cowen & Sponaugle, 2009). Theoretically, given sufficient prey availability in planktotrophic larvae, more rapid growth has the potential to improve survival of larval fish; larvae could more rapidly outgrow gape‐limited predators. As basal metabolic rates, growth, development and energetic costs of larvae are determined in general by water temperature, increasing ocean temperatures will generally reduce the duration of the larval phase (PLD) (O'Connor et al., 2007), and thus decrease the cumulative mortality risk during this particularly vulnerable life phase. However, such effects are offset by the theoretical increase in instantaneous mortality at higher temperatures due to starvation if nonfeeding, or if food availability and feeding become uncoupled (Kristiansen, Drinkwater, Lough, & Sundby, 2011). A shorter PLD also implies that larval fish may no longer have sufficient time to disperse between scattered habitats, and a higher proportion die in the open ocean before they can reach a suitable settlement site (Kendall, Poti, Wynne, Kinlan, & Bauer, 2013; O'Connor et al., 2007).

In addition to biological effects, a warmer climate may change underlying ocean conditions. Increased vertical stratification caused by stronger surface heat fluxes and increased freshwater input from precipitation and ice melt may impact not only primary productivity, with consequences for the availability of prey, but also vertical mixing and patterns of circulation (Meijers, 2014). It has been theorized that warming in the Southern Ocean over recent decades is in part due to a poleward shift of the frontal features of the Antarctic Circumpolar Current (e.g., Boning, Dispert, Visbeck, Rintoul, & Schwarzkopf, 2008; Gille, 2008), though whether such a shift has occurred is subject to debate, with recent assessments casting doubt on this (e.g., Gille, 2014). The strength and direction of currents may also be influenced by large‐scale modes of climate variability, such as El Niño‐Southern Oscillation and the Southern Annular Mode, although the response is likely to be complex and involve interactions between the mean flow and eddy field (e.g., Hallberg & Gnanadesikan, 2006). Overall, there is currently considerable uncertainty concerning the impact of climate change on circulation patterns in the Southern Ocean, and no clear consensus across the latest generation of coupled climate models (Meijers, 2014).

Identifying key drivers that influence marine connectivity is especially pertinent in Antarctic waters that are experiencing unprecedented rates of warming (Meredith & King, 2005; Whitehouse et al., 2008), and where historical localized collapses of exploited commercial fishes increase vulnerability to marked shifts in trophic relations (Kock, 1992; Layman, Quattrochi, Peyer, & Allgeier, 2007). Moreover, the link between dispersal and gene flow depends ultimately on successful reproduction of migrants in recipient populations and phenotypic match of the offspring to the local environment, and thus is not necessarily a function of immigrant larval recruitment. In particular, the inference of population connectivity from genetic data alone can be misleading, primarily because of variance in effective population size and nonequilibrium conditions on estimates of the fixation index, *F*_*ST*_ (Faurby & Barber, 2012; Waples & Gaggiotti, 2006). It follows, therefore, that a multidisciplinary approach to exploring marine connectivity is optimal to capture the diversity of processes underway.

Here, we employ data and inferences from life histories, physiology, population genetics and oceanography, supported through simulations, to examine the likely impact of predicted shifts in Antarctic water temperatures on dispersal, gene flow and population genetic structure. Specifically, we use an established modelling system (Young et al., 2015) to investigate how changes in the duration of the planktonic phases and mortality rates of two fish species with contrasting life histories may affect connectivity and population genetic structure within the Scotia Sea. We then consider the wider implications of our findings for the resilience of fragmented populations under climate change scenarios.

## 2 MATERIALS AND METHODS

### 2.1 Study region

**Figure 1:**
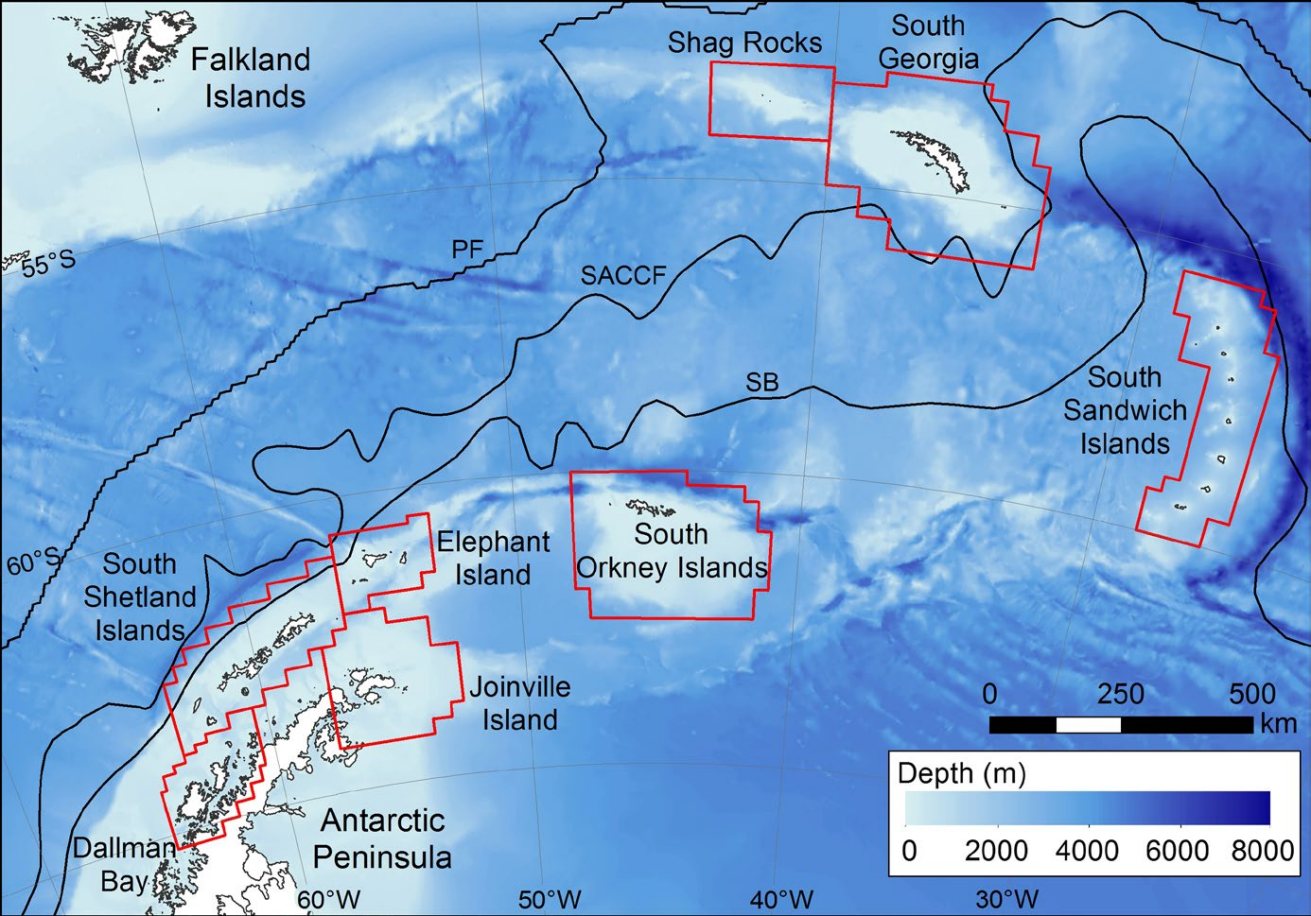
Map of the Scotia Sea region. Population boxes used in the connectivity analyses are outlined in red. Thin black lines indicate the mean positions of the Polar Front (PF) following Moore, Abbott, and Richman (1997), the Southern ACC Front (SACCF) and the southern boundary of the ACC (SB), both from Thorpe (2001). Bathymetry is from the GEBCO_2014 grid, version 20150318 (http://www.gebco.net)

We focus on the Scotia Sea (Figure 1), which is one of the most productive regions in the Southern Ocean supporting high abundances of zooplankton, fish, seabirds and marine mammals (Murphy et al., 2007). This region has recently experienced rapid increases in ocean temperatures, with surface summer temperatures near the Western Antarctic Peninsula increasing by >1°C in the latter half of the 20th century (Meredith & King, 2005), and increases in wintertime near‐surface temperatures near South Georgia of ~2.3°C for the period 1925–2006 (Whitehouse et al., 2008). Further significant warming of up to 2.5°C by the end of the 21st century is predicted (Figure S1). Thus, it is a prime location for considering the response of population connectivity and resilience to climate change. Horizontal flow in this region is dominated by the Antarctic Circumpolar Current (ACC), which is known to have been broadly stable since the Last Glacial Maximum (McCave, Crowhurst, Kuhn, Hillenbrand, & Meredith, 2014), with only relatively low levels of shorter period variability superposed (e.g., Meredith, Woodworth, Hughes, & Stepanov, 2004). Such long‐term stability suggests that populations are likely to have reached migration–drift equilibria (Wright, 1931), which lends credence to the baseline present‐day genetic projection modelling utilized in our study. The ACC has a banded structure consisting of several fast‐moving current jets separated by relatively quiescent zones of water (Orsi, Whitworth, & Nowlin, 1995). Each of these jets is associated with a frontal region characterized by marked meridional gradients in temperature and/or salinity. Within the Scotia Sea, the ACC flows in a predominantly north‐eastward direction, but is also characterized by strong mesoscale activity, whereby instabilities in the current cores lead to frequent generation of eddies, with spatial scales up to tens of kilometres. Thus, whilst the dominant north‐eastward flows have a major role in connecting populations and food webs, transporting organisms from areas around the Antarctic Peninsula north and east across the Scotia Sea (Matschiner, Hanel, & Salzburger, 2009; Murphy, Thorpe, Watkins, & Hewitt, 2004), previous modelling work has demonstrated spatial and inter‐annual variability in larval dispersal (Young et al., 2015).

### 2.2 Study species

We focus on populations of two Antarctic fish species within the Scotia Sea, *Champsocephalus gunnari* and *Notothenia rossii*. These species were chosen based on their relatively isolated shelf habitats and salient differences in respective early life histories. The two species share similar distribution patterns, with populations around sub‐Antarctic islands and at the northern end of the Antarctic Peninsula (e.g., Gon & Heemstra, 1990; Kock & Everson, 2003), although Shag Rocks does not have a spawning population of *N. rossii*. However, whilst *C. gunnari* spawns benthic adhesive eggs (Everson et al., 2001) in deep (100–350 m) inshore waters and fjords (Frolkina, 2002; Kock, 2005), *N. rossii* spawns planktonic eggs in offshore shelf areas with depths of around 200–360 m (Kock, Belchier, & Jones, 2004). The incubation period of *C. gunnari* eggs is approximately 4 months at South Georgia and Shag Rocks (North, 2001), increasing to around 6 months at more southerly locations (Kock, 2005). After hatching, the larvae spend approximately 3 months dispersing passively with the ocean currents (Duhamel, 1995), vertically distributed within the upper 50 m of the water column (North & Murray, 1992), before developing into juveniles. At this stage, they are able to undergo considerable diurnal vertical migration and can no longer be considered planktonic. For the first two years of life, *C. gunnari* use the shallow shelf areas as a nursery ground (Duhamel, 1995). *N. rossii* eggs develop into larvae after approximately 4 months in the Scotia Sea (Kock & Kellermann, 1991), and then into blue fingerlings after about a further 3 months, at which stage they migrate inshore to kelp beds (Gon & Heemstra, 1990). *N. rossii* eggs are found in the top 100 m of the water column, and their larvae in the upper 50 m (*North*, pers. comm.). The total planktonic phase of *N. rossii* is thus more than double that of *C. gunnari*, and it is this key characteristic of their early life histories that drives differences in patterns of population differentiation; with a shorter planktonic phase and reduced dispersal capability, *C. gunnari* exhibits greater genetic structuring across the Scotia Sea (Young et al., 2015).

### 2.3 Individual‐based model

The underlying modelling techniques used in this study are described in detail by Young et al. (2015); here we provide a summary of the numerical models and highlight the changes made to the parameterization and implementation of the models for the climate change simulations.

Five‐day mean velocity fields from a state‐of‐the‐art oceanographic modelling framework, NEMO (Nucleus for European Modelling of the Ocean), provided by the National Oceanography Centre, Southampton, were used to advect Lagrangian particles representing the early life stages of *C. gunnari* and *N. rossii*. NEMO has been widely used over a range of spatial scales and resolutions and has been shown to provide a good representation of the dominant oceanography of the Antarctic Peninsula and Scotia Sea region (Renner, Heywood, & Thorpe, 2009; Renner et al., 2012). Full details may be found at http://www.nemo-ocean.eu/About-NEMO with the specific NEMO application used in this study described by Young et al. (2015). The Lagrangian model has been parameterized for the simulation of *C. gunnari* and *N. rossii* eggs and larvae (Young et al., 2012), and its application to the study of the present‐day population connectivity of these fish species in the Scotia Sea has been demonstrated (Young et al., 2015). In summary, particles are advected at each model time step (5 min) according to the imposed three‐dimensional velocity field, using a second‐order Runge‐Kutta method. Additional horizontal and vertical diffusions are included using a random‐walk approach (Dyke, 2001).

The reference present‐day simulations are described by Young et al. (2015). The representative study period, 1996–2001, includes years with relative extremes in atmospheric forcing over the Southern Ocean, associated with extreme phases of large‐scale coupled modes of inter‐annual climate variability (see Meredith, Murphy, Hawker, King, & Wallace, 2008; for full discussion). This choice of study period allows robust inferences concerning larval dispersal whilst accounting for variability in present‐day physical forcing (Young et al., 2015). For each of the five study years, model particles representing the early life stages of the two fish species were released at the locations of known spawning populations described in published literature (population boxes; Figure 1; Barrera‐Oro & Casaux, 1992; Everson et al., 2001; Frolkina, 2002; Kock et al., 2004; Parkes, 2000). 1,000 particles were released per day at each site for the duration of the spawning periods, with species‐specific characteristics assigned to each particle (for full details, see Young et al., 2015). Pairwise transport matrices ***TM*** for each species were then constructed, describing the proportion of individuals arriving in a destination population (rows) from a given source population (columns), and allowing a 4‐week recruitment window at the end of the prescribed planktonic period. These were converted to connectivity matrices ***M*** by incorporating egg and larval mortality, assuming temperature‐dependent mortality rates from Pepin (1991),

(1) $$\begin{array}{cc} z_{e} & =0.030e^{0.18T}\;,\;\text{for}\;\text{eggs}\\ & z_{l}=0.044e^{0.077T}\;,\;\text{for}\;\text{larvae} \end{array}$$

with an assumed present‐day temperature (*T*) of 1°C, representative of annual mean near‐surface sea temperatures across the Scotia Sea (Mapping and Geographic Information, British Antarctic Survey, pers. comm.).

### 2.4 Projected changes to early life histories

The baseline present‐day simulations were repeated for a future scenario of increased temperatures. A rise in sea surface temperature of 2.5°C was assumed, which is within the range of the forecast increase in temperature for the Scotia Sea region by the end of the 21st century from the latest generation of coupled climate model simulations (Figure S1).

*Notothenia rossii* has pelagic egg and larval stages, and thus, the same temperature increase was assumed to impact both the eggs and larvae. However, *C. gunnari* lays benthic eggs and it is difficult to predict with certainty how climate change may impact near‐bed shelf temperatures, in part due to the poor resolution of global models used for climate change simulations, and their inadequate representation of shelf regions. However, analyses of recent temperature changes at the Antarctic Peninsula suggest that near‐bed temperatures may well increase less than near‐surface temperatures (Meredith & King, 2005). Thus, for *C. gunnari*, the increase in sea surface temperatures was assumed to impact only the larval phase. The effect of a possible increase in near‐bed temperatures on the egg phase was investigated through a series of sensitivity experiments (see *Model sensitivity study*).

We considered two physiological responses to increased temperatures in this study: egg and larval stage durations and mortality rates. Relative to the present‐day simulations, a reduction in the duration of the egg phase was determined using equations detailed by Hirst and Lopez‐Urrutia (2006), and data on marine fish egg development in Pauly and Pullin (1988) (Appendix A). The duration of the larval phases was shortened following the universal model for the temperature dependency of larval development of marine animals developed by O'Connor et al. (2007). The effect of increased temperature on egg and larval mortality rates was estimated using the generalized temperature‐dependent mortality rates from Pepin (1991; Equation (1)). The subsequent present‐day and climate change model parameterizations are detailed in Table 1. Changes to the species‐specific pairwise transport and connectivity matrices were calculated, firstly considering solely the effect of shortened planktonic phase and secondly considering changes to both planktonic phase and mortality rates.

**Table 1:** Model parameterization for the present‐day and climate change simulations. Egg durations for *Champsocephalus gunnari* are for the southern/northern populations in the Scotia Sea, respectively

	*N. rossii*	*C. gunnari*
Egg duration (days)		
Present day	120	180/120
+2.5°C	93	180/120
Larval duration (days)		
Present day	90	90
+2.5°C	72	72
Egg mortality (per day)		
Present day	0.0359	0.0359
+2.5°C	0.0563	0.0359
Larval mortality (per day)		
Present day	0.0475	0.0475
+2.5°C	0.0576	0.0576

### 2.5 Population genetic structure projection model

The methodology used to project population genetic structure is described in detail by Kool, Paris, Andrefouet, and Cowen (2010), and Kool, Paris, Barber, and Cowen (2011), and the application of the methodology to understand observed present‐day patterns of population genetic structure in *C. gunnari* and *N. rossii* populations is described by Young et al. (2015). Briefly, a connectivity matrix ***M*** is applied to a state matrix ***Q***_*t*_ containing information on the frequency of alleles of each type in each population at time *t*, to yield the expected state of the population at time *t *+* *1. Thus,

(2) $$Q_{t+1}=\left(\bar{{MBQ}_{t}+Q_{t}}\right)$$

with ***B*** a diagonal matrix of birth rates for each population, and the top bar indicating row normalization. The connectivity matrix, ***M***, is generated by the individual‐based model and thus varies by species and climate change parameterization. Using the assumption that individuals are uniquely labelled according to their population of origin by initially fixing populations for different alleles, ***Q***_0_ = ***K***, with ***K*** a diagonal matrix of carrying capacity values for each population. Data on population sizes of the fish species at each spawning site are sparse, thus the carrying capacities of each site were assumed to be the same. Mean birth rates were derived from empirical fecundity data (Kock & Kellermann, 1991), with 2,800 larvae per individual *C. gunnari* and 20,000 larvae per individual *N. rossii*.

Expected genetic connectivity for each ***M*** was projected forward in time to generate estimates of allele frequencies for each population (Figure 1). Pairwise heterozygosity standardized fixation indices, *G”*_*ST*_, were calculated at each time step using equations from Meirmans and Hedrick (2011) for visualization of the projected genetic structure. For the present‐day projections described by Young et al. (2015), the model was halted once the maximum projected *G”*_*ST*_ reached the maximum observed value from empirical microsatellite data. For the future projections, two cut‐off points were used to understand the potential impacts of warmer sea temperatures on genetic structure. Firstly, the model was halted after the number of time steps required by the present‐day projection to reach the observed levels of genetic structure, estimated in Young et al. (2015). By comparing like‐with‐like, this approach illustrates the potential impacts of climate change referenced to present‐day time scales. Secondly, as with the present‐day projection (Young et al., 2015), the model was halted once the maximum projected *G”*_*ST*_ reached the maximum observed empirical value. In this way, we measure the difference in time steps that it takes the model under the climate change scenario to reach current empirical levels of population differentiation and explore the development of the projected population genetic structure.

As in Young et al. (2015), discriminant analysis of principal components (DAPC; Jombart, Devillard, & Balloux, 2010) in the ADEGENET package (Jombart, 2008) for R (R 3.4.1; R Core Team (2017)), visualized patterns of genetic structuring among sites of both species for simulated connectivity under present‐day and future climate change conditions. As most information on genetic structuring was contained in the first principal component, and to ease visualization of population differentiation, only the density of individuals belonging to each site was plotted along the first discriminant function.

### 2.6 Model sensitivity study

The effect of a possible increase in near‐bed temperatures on the egg phase of *C. gunnari* was investigated through a series of sensitivity experiments, with assumed near‐bed temperature increases of 0.5, 1 and 1.5°C. For each, larval phase was assumed to experience a rise of 2.5°C in near‐surface temperatures. The range of model parameterizations for these sensitivity studies is detailed in Table 2.

**Table 2:** Model parameterizations for the *Champsocephalus gunnari* sensitivity study with increased near‐bed temperatures of +0.5, +1.0 and +1.5°C. Egg durations are for the southern/northern populations in the Scotia Sea, respectively

	+0.5°C	+1.0°C	+1.5°C
Egg duration (days)	171/114	162/108	154/103
Larval duration (days)	72	72	72
Egg mortality (per day)	0.0393	0.0430	0.0470
Larval mortality (per day)	0.0576	0.0576	0.0576

## 3 RESULTS

### 3.1 Transport and connectivity

**Figure 2:**
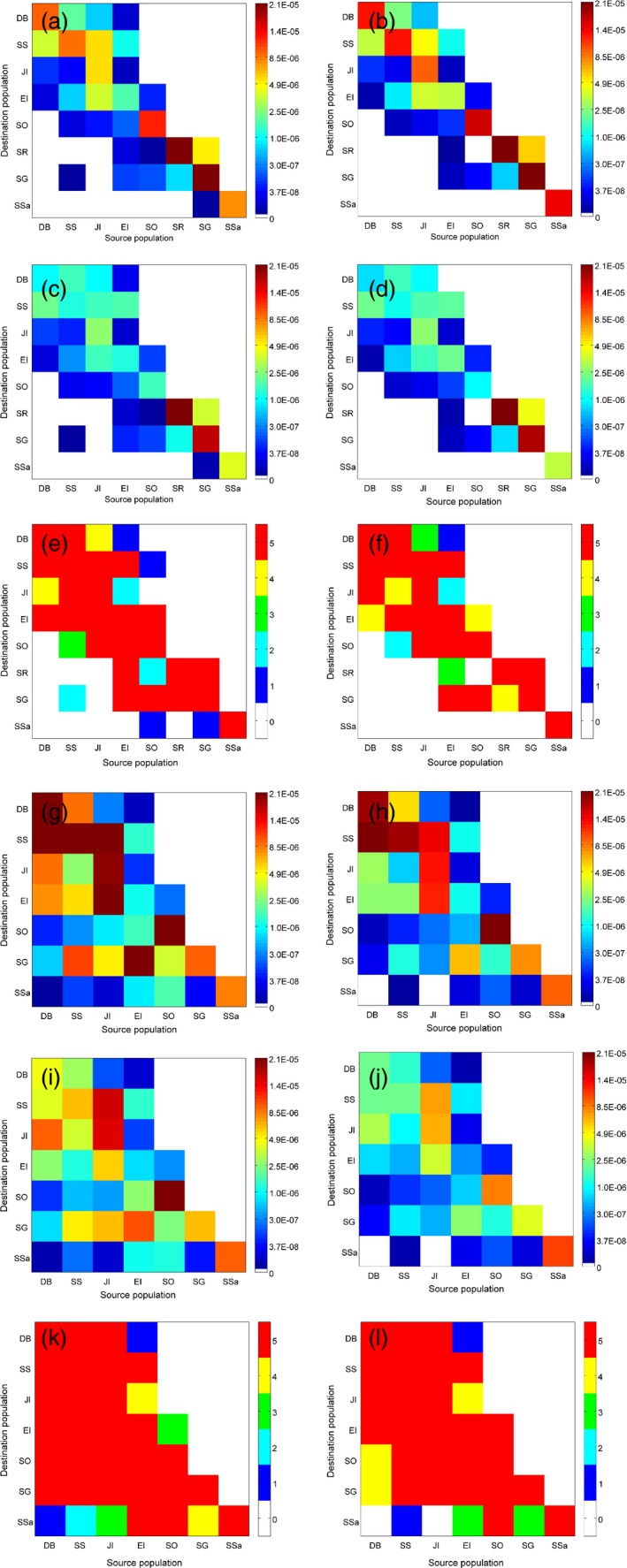
Patterns of connectivity for *Champsocephalus gunnari* (a–f) and *Notothenia rossii* (g–k) for the present day (a, c, e, g, i, k) and under a climate change scenario of +2.5°C (b, d, f, h, j, l): mean (a, b, g, h), standard deviation (c, d, i, j) and frequency of nonzero connectivity (e, f, k, l). Population identifiers are Dallman Bay (DB), South Shetland Islands (SS), Joinville Island (JI), Elephant Island (EI), South Orkney Islands (SO), Shag Rocks (SR), South Georgia (SG) and South Sandwich Islands (SSa); see Figure 1 for locations. Note, the colour scales in (a–d) and (g–j) are transformed to allow visualization of the full range of values

Present‐day patterns of connectivity indicated wider dispersal of *N. rossii* than of *C. gunnari*, with more consistent and higher mean levels of *N. rossii* transport across the Scotia Sea to islands in the north and east (South Georgia and South Sandwich Islands) relative to *C. gunnari* (Figure 2). Dispersal was highly asymmetric for both species, with higher values below the diagonals of the connectivity matrices, indicating uni‐directional transport to the northeast across the Scotia Sea in accordance with the dominant north‐eastward flows of the Antarctic Circumpolar Current. However, complex oceanographic flows around the Antarctic Peninsula and near South Georgia and Shag Rocks generated bi‐directional transport between sites in these regions. There was notable variability in levels of self‐recruitment among sites, indicated by values along the diagonal (Figure 2), with consistently low levels of self‐recruitment at Elephant Island for both species. Self‐recruitment was generally stronger for *N. rossii* than *C. gunnari*, with the exception of South Georgia, but with high levels of inter‐annual variability suggested by the standard deviations (Figure 2i).

**Figure 3:**
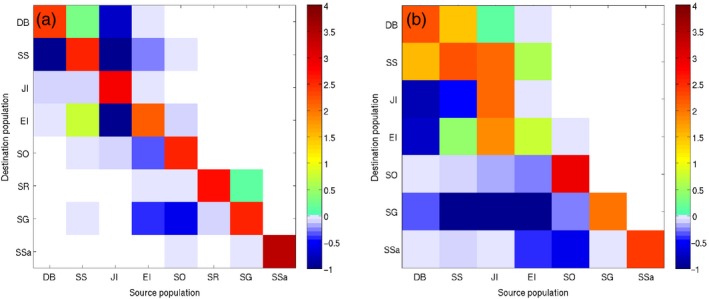
Predicted changes to pairwise transport with increased sea temperatures (increased temperature scenario minus present day, expressed as the percentage of released particles on a transformed log scale to allow visualization of the full range of values) for *Champsocephalus gunnari* (a) and *Notothenia rossii* (b). Population identifiers are Dallman Bay (DB), South Shetland Islands (SS), Joinville Island (JI), Elephant Island (EI), South Orkney Islands (SO), Shag Rocks (SR), South Georgia (SG) and South Sandwich Islands (SSa); see Figure 1 for locations

A rise in sea surface temperatures of 2.5°C was predicted to reduce the planktonic phases of *N. rossii* and *C. gunnari* from 210/90 days to 165/72 days, respectively (Table 1). Neglecting mortality and considering solely the impact of the shorter planktonic phases on pairwise transport, predictions suggested a decrease in transport across the Scotia Sea from the southern Scotia Arc to South Georgia and the South Sandwich Islands for both species, to Shag Rocks for *C. gunnari*, and a decrease in transport from the Antarctic Peninsula to the South Orkney Islands (Figure 3). The shorter planktonic phases also generated an increase in local retention at all spawning sites, and an increase in transport between neighbouring South Georgia and Shag Rocks (*C. gunnari*). However, the complexity of oceanography in the Antarctic Peninsula region generated a variable response to the shortening of the planktonic phases. Whilst the model simulations predicted a general increase in pairwise transport for *N. rossii* around the Antarctic Peninsula, reduced transport was predicted to Joinville Island from Dallman Bay and South Shetland Islands, and from Dallman Bay to Elephant Island. By contrast, predictions for *C. gunnari* showed a general decrease in pairwise transport, except for transport from the South Shetland Islands to Elephant Island and Dallman Bay.

Instantaneous egg and larval mortality rates were predicted to increase with higher sea temperatures (Equation (1); Table 1). However, the reduction in the length of the planktonic phases modulated the impact of this increase on the integrated mortality. Thus, whilst integrated mortality during the egg phase of *N. rossii* increased, integrated mortality of the larval phases of both species decreased slightly. Consequently, overall survival of *N. rossii* decreased, but for *C. gunnari* it increased. The new mortality rates were used to derive connectivity matrices from the simulated pairwise transport patterns (Figure 2). For *N. rossii*, the reduced connectivity across the Scotia Sea due to a shorter planktonic period was further reduced by the increased mortality rates in the climate change simulation. Over shorter geographic distances, where connectivity and local retention were broadly predicted to increase with the shorter planktonic period, the increased mortality rates were sufficient to negate this effect, with the exception of South Sandwich Islands. Although *C. gunnari* was predicted to have a slight increase in integrated survival under warmer climate conditions, this was not sufficient to modify significantly the changes in connectivity due to the shorter planktonic period. Thus, there was a reduction in exchange across the Scotia Sea, an increase in local retention, a small increase in connectivity between South Georgia and Shag Rocks and an overall decrease between the majority of site pairs around the Antarctic Peninsula.

**Figure 4:**
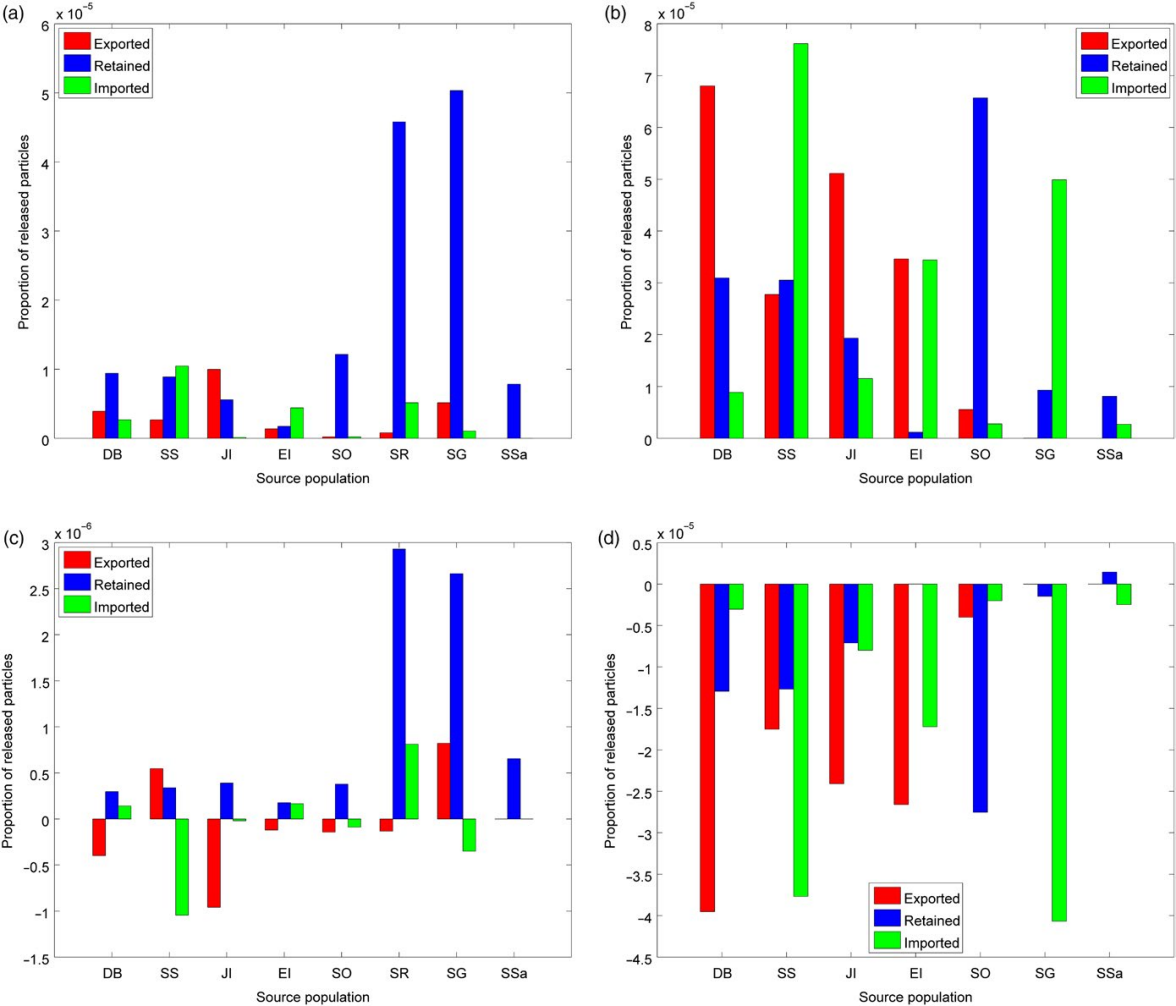
Proportion of released particles that are exported, imported and retained at sites across the Scotia Sea in simulations of present‐day connectivity of *Champsocephalus gunnari* (a) and *Notothenia rossii* (b), and the change in these quantities for a rise in sea temperatures of 2.5°C (*C. gunnari*: c; *N. rossii*: d; increased temperature simulation minus present day). Site identifiers are Dallman Bay (DB), South Shetland Islands (SS), Joinville Island (JI), Elephant Island (EI), South Orkney Islands (SO), Shag Rocks (SR), South Georgia (SG) and South Sandwich Islands (SSa); see Figure 1 for locations. Note, *y*‐axis scales are not the same, and “retained” in (c) is scaled by a factor of 10^−1^

The relative roles of individual sites as net larval importers or exporters, and how this may change with rising sea temperatures, were compared for *C. gunnari* and *N. rossii* (Figure 4). In the present day, the sites with the greatest levels of export were located around the Antarctic Peninsula for both species, in particular Joinville Island and Dallman Bay, with equivalent levels of exchange between South Georgia and Shag Rocks also predicted for *C. gunnari*. The dominant importers of larvae around the Antarctic Peninsula were South Shetland Islands and Elephant Island. A large proportion of *N. rossii* larvae were transported across the Scotia Sea and imported to the population at South Georgia, where the proportion of imported larvae exceeded that retained locally. However, *C. gunnari* at South Georgia and neighbouring Shag Rocks were strongly reliant on retention of local spawning, as were populations of both species at South Sandwich and South Orkney Islands. With warmer sea temperatures, the broad pattern of import and export sites remained similar. However, successful export from sites around the Antarctic Peninsula and South Orkney Islands was predicted to decrease, with a corresponding decrease in imports at sites downstream, which particularly impacted *N. rossii* at South Georgia and South Sandwich Islands. For both species, local retention constituted a greater proportion of overall recruitment at all sites, but in particular at South Georgia, South Sandwich Islands, South Orkney Islands and Shag Rocks (*C. gunnari*).

**Figure 5:**
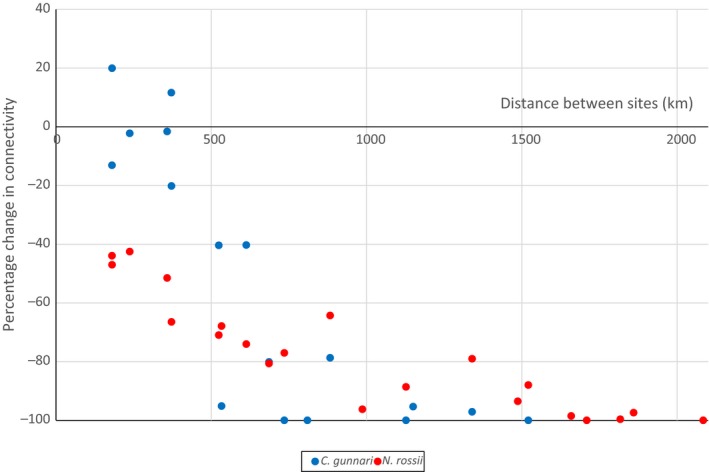
Percentage change in connectivity due to increased sea temperatures against distance between sites, for *Champsocephalus gunnari* and *Notothenia rossii*

The correlation of predicted pairwise connectivity with geographic (Euclidean) distance between sites was assessed using Mantel tests. A significant negative correlation was found for the present day for *C. gunnari* (*r* = −.60; *p* < .001), with a weaker negative correlation for *N. rossii* (*r* = −.40; *p* = .048). Increasing sea temperatures by 2.5°C had little impact on the correlation for *C. gunnari* (*r* = −.59; *p* < .001) and increased the strength of the negative correlation for *N. rossii* (*r* = −.49; *p* = .013). Thus, the reduced planktonic phase of *N. rossii* and subsequent weaker dispersal potential increased the impact of site separation on connectivity of *N. rossii*. For both species, the percentage change in pairwise connectivity due to increased temperatures was greater for more distant sites, with connectivity reduced by more than 90% for most sites separated by greater than ~1,000 km (Figure 5), implying that increased temperatures had a relatively greater impact on connectivity between more distant sites. Significant negative correlations were predicted for the percentage change in connectivity with distance between sites for both species, with correlation coefficients of *r* = −.82 (*p* = 5 × 10^−5^; *C. gunnari*) and *r* = −.89 (*p* = 3 × 10^−8^; *N. rossii*).

### 3.2 Population genetic structure

**Figure 6:**
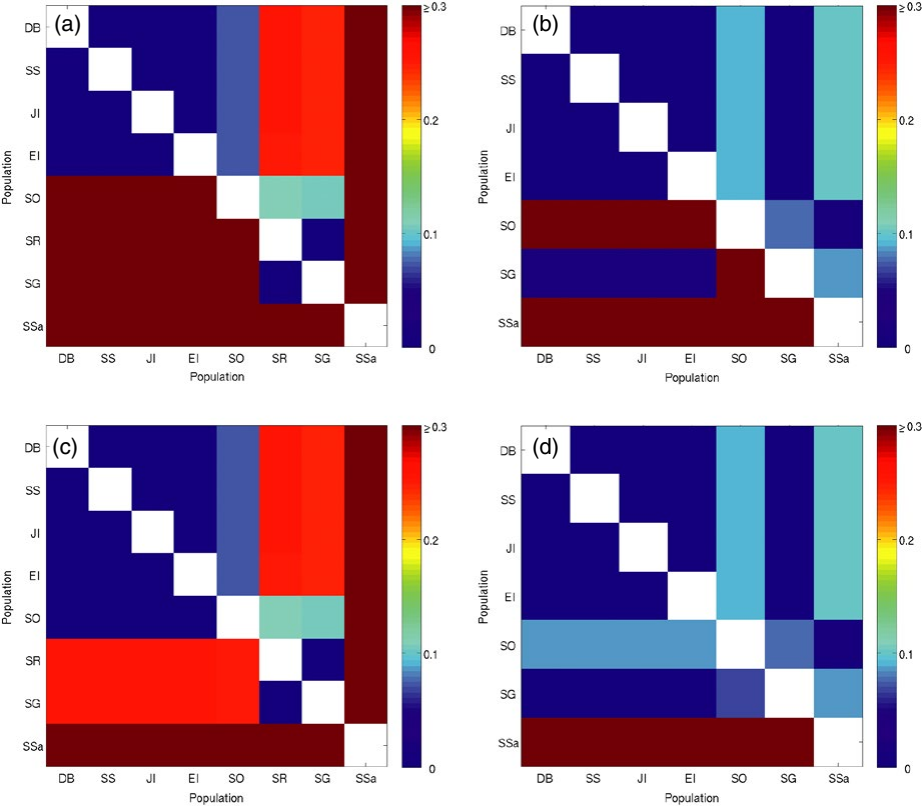
Projected genetic differentiation between populations (*G”* _*ST*_) for *Champsocephalus gunnari* (a, c) and *Notothenia rossii* (b, d); present day above the diagonal, increased temperature scenario below the diagonal. Model projections were stopped after the same number of time steps as the present‐day simulations (a, b), or once the maximum projected genetic differentiation reached present‐day levels (c, d)

**Figure 7:**
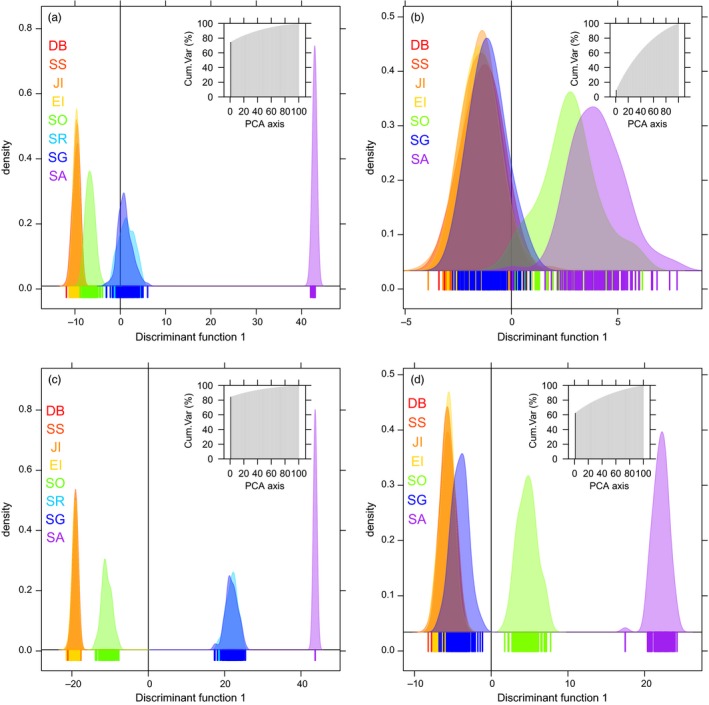
Discriminant analysis of principal components plots of the simulated genetic structure among populations for *Champsocephalus gunnari* (a, c) and *Notothenia rossii* (b, d); scatter plots of the first principal component illustrating the density of simulated individuals along the first discriminant function for present‐day connectivity simulations (a, b) and connectivity simulations under the climate change scenario (c, d). The | along the x axis represent simulated individuals, the different colours represent sites: Dallman Bay (DB), South Shetland Islands (SS), Joinville Island (JI), Elephant Island (EI), South Orkney Islands (SO), Shag Rocks (SR), South Georgia (SG) and South Sandwich Islands (SA). The number of principal components retained (1 in every case), and the cumulative variance explained are highlighted in black in the inset. Note: the climate change simulation resulted in no connectivity between SA and the other sites for *C. gunnari*, and hence, all 100 individuals are genetically monomorphic and there are no shared alleles between SA and the other sites, resulting in a single | in c

Projections of present‐day population genetic structure derived from the connectivity matrices differed considerably between the two species (Figure 6, above diagonals; Figure 7a,b) and were in good agreement with empirically derived patterns of genetic structuring from prior microsatellite analyses (Young et al., 2015). Marked genetic structuring was projected for *C. gunnari*, with four groupings identified: (i) the Antarctic Peninsula region, (ii) South Orkney Islands, (iii) South Georgia and Shag Rocks and (iv) South Sandwich Islands (Figure 7a). By contrast, the projected level of population genetic structuring for *N. rossii* was markedly weaker with only two groupings suggested: (i) the Antarctic Peninsula region and South Georgia and (ii) South Orkney and South Sandwich Islands (Figure 7b). The projected structure of both species was influenced by stepping stone transport, but acting over different spatial scales. For *C. gunnari*, transport from the Antarctic Peninsula to South Georgia and Shag Rocks was achieved via Elephant Island and South Orkney Islands, and the South Sandwich Islands were strongly isolated, with only weak and intermittent connectivity with South Georgia and South Orkney Islands (Figure 2). By contrast, consistent direct transport from the Antarctic Peninsula to South Georgia resulted in weak genetic differentiation between these sites for *N. rossii*, and stepping stone transport was important at larger geographic scales, in particular across the Scotia Sea to the South Sandwich Islands via South Orkney Islands. As a consequence, correlations of projected *G”*_*ST*_ against Euclidian distance between source and destination sites revealed a significant isolation‐by‐distance pattern in *C. gunnari* (*r* = .89; *p* < .001), but not in *N. rossii* (*r* = −.36; *p* = .49). The number of time steps required for the projected maximum *G”*_*ST*_ to reach observed levels of differentiation was considerably fewer for *N. rossii* (83) than *C. gunnari* (3519); such difference between species coincides with the expected faster approach to equilibrium in populations connected by higher migration rates (Whitlock & McCauley, 1999).

The population genetic projections were repeated using the predicted connectivity matrices for an increased temperature of 2.5°C (Figure 2b,h), first halting the simulations after 83 (*N. rossii*) or 3519 (*C. gunnari*) time steps, as for the present‐day projections (Young et al., 2015). For both species, low genetic differentiation persisted among sites in the Antarctic Peninsula region (Figure 6a,b; below diagonal). However, *N. rossii* at the South Orkney and South Sandwich Islands were predicted to become much more isolated from each other and from the Antarctic Peninsula, as illustrated in the DAPC plots (Figure 7d), with high genetic differentiation between these and all other sites within the Scotia Sea. Furthermore, the reduction in connectivity between Antarctic Peninsula sites and South Georgia for *N. rossii* leads to a slight emergence of genetic structuring between them on the DAPC plots (Figure 7d). Similarly, *C. gunnari* sites away from the Antarctic Peninsula region were predicted to become considerably more isolated from each other (Figure 7c), with the exception of continued genetic homogeneity between South Georgia and Shag Rocks (Figures 6a and 7c). These projections suggest that, referenced to present‐day time scales, populations of both *N. rossii* and *C. gunnari* in the Scotia Sea would become more genetically fragmented and isolated as temperature increases. This is particularly marked for *C. gunnari* populations, with high genetic differentiation projected for all but the most proximate sites.

The potential for populations of *C. gunnari* and *N. rossii* under warmer temperatures to reach present‐day levels of genetic differentiation was assessed by running the increased temperature genetic projections until the maximum *G”*_*ST*_ reached the maximum observed value from empirical microsatellite data. These projections required many more time steps to complete: 2.5/5.6 times the number of time steps compared to the present day for *N. rossii* and *C. gunnari*, respectively. However, although projections for *N. rossii* took longer to approach present‐day levels of genetic differentiation, there was sufficient gene flow to maintain broad genetic homogeneity over relatively short timescales (211 time steps), albeit with a notable increase in isolation of the population at the South Sandwich Islands (Figure 6d). Projections suggested that genetic differentiation between northern and southern populations of *C. gunnari* would eventually approach present‐day levels, with the population at South Orkneys becoming genetically more similar to that at the Antarctic Peninsula (Figure 6c). However, the significant increase in the number of time steps required to reach this point suggests that increased regional isolation is more likely.

### 3.3 Model sensitivity study

The control run for comparison with the sensitivity experiments was the climate change simulation described above, with larval duration and mortality for an assumed temperature increase of +2.5°C. In the first series of experiments, the effect of increasing temperature on egg duration was considered, with the egg mortality rate held at present‐day levels. In the second series of experiments, the effects of increasing temperature on both egg duration and mortality rate were included.

Theoretically, egg duration decreases as temperature increases (Appendix A; Table 2), and with egg mortality fixed at present‐day levels, increased percentage survival at the end of the egg phase was predicted. As a result, pairwise connectivity and local retention increased with benthic temperature. However, although the number of time steps required to reach present‐day levels of genetic differentiation decreased with increasing benthic temperature (from 19,690 to 8,657 for a temperature increase of 1.5°C), the projected population genetic structure within the Scotia Sea was virtually identical to that from the control climate change simulation.

Instantaneous egg mortality rates were predicted to increase with warmer sea temperatures (Equation (1); Table 2) and, although egg durations were shorter, integrated mortality over the egg phase increased with temperature. Consequently, pairwise connectivity and local retention decreased with increasing benthic temperature and the number of time steps required to reach present‐day levels of genetic differentiation increased (from 19,690 to 42,290 for a benthic temperature rise of 1.5°C). This suggests that the predicted reduction in connectivity between populations arising from increased sea surface temperatures will occur more rapidly if benthic temperatures also increase. However, as with the first series of sensitivity experiments, the projected population genetic structure within the Scotia Sea at the end of the simulation was virtually identical to that from the control run.

These sensitivity experiments demonstrated that including an increase in benthic temperature in the model simulations through variability in egg duration and mortality did not affect projected population genetic structure of *C. gunnari* in the Scotia Sea. However, it had a significant impact on the number of model time steps required for the projections to approach present‐day levels of genetic differentiation. Therefore, although these levels of differentiation were eventually reached, the results suggest that changes to the duration of the egg phase and egg mortality due to rising temperatures would further increase isolation of northern Scotia Sea populations, with a significant impact on population genetic structure.

## 4 DISCUSSION

### 4.1 Shifts in population connectivity

With a rise in sea temperatures, the theoretical shortening of the planktonic phases of *C. gunnari* and *N. rossii* is predicted to reduce transport between southern and northern populations in the Scotia Sea. Over smaller scales, the two species are predicted to respond somewhat differently to increased temperatures. Broadly, there is a tendency towards a decrease in pairwise transport between sites around the Antarctic Peninsula for *C. gunnari*, but an increase for *N. rossii*. However, once increases in the mortality rates of eggs and larvae due to the rise in sea temperatures are incorporated, there is a decrease in connectivity between all *N. rossii* populations and the majority of *C. gunnari* populations. Notably, the frequency of connectivity of many site pairs is reduced implying both lower magnitude and less consistent connectivity. Although many of the predicted changes in connectivity are relatively small (Figure 2), they may have demographic and evolutionary significance over time (Kendall, Poti, & Karnauskas, 2016) and will depend critically on local effective population size, extent of local adaptation, and rate and magnitude of environmental change, especially extreme episodic events such as El Ninõ (Wood et al., 2016). A progressive reduction in dispersal and gene flow, for example, over long timescales constrains population resilience and sustainability, especially in exploited fish populations where recovery from over‐fishing is slow (Murawski, 2010). Both *C. gunnari* and *N. rossii* were heavily exploited across their range in the 1970s and 1980s (Ainley & Blight, 2009). Although bans on targeted fishing of both species were introduced by CCAMLR in the late 1980s, recent trawl estimates of spawning stock biomass suggest populations of both species across the Scotia Sea remain below pre‐exploitation levels (E. Barrera‐Oro, Marschoff, & Ainley, 2017).

As recently emphasized in a meta‐analysis of ecological attributes promoting ecological resilience (Timpane‐Padgham, Beechie, & Klinger, 2017), connectivity among populations is a major factor promoting genetic diversity, resilience to disturbance and maintenance of ecosystem‐level processes such as productivity and food webs. Compared to tropical and temperate waters, polar seas are frequently dominated by relatively few top predators and somewhat simpler trophic relationships. When krill, *Euphausia superba,* is abundant, it dominates the diet of both *C. gunnari* and *N. rossii*, although they can switch to alternative prey types during periods of low krill abundance (Main, Collins, Mitchell, & Belchier, 2009). As trophic niche width of key predators is predicted to reduce with habitat fragmentation (Layman et al., 2007; Timpane‐Padgham et al., 2017), driven by the lower diversity in prey items, such shifts can exert consequences at the ecosystem level. Reduced trophic niche width can destabilize local food webs and correspondingly increase susceptibility of predators to extinction through time. Here, such effects are likely to be amplified by reductions in fish population size and ongoing habitat change.

### 4.2 Patterns of larval supply

Populations of *N. rossii* around the Antarctic Peninsula are important exporters of eggs and larvae to populations to the north and east, in particular to South Georgia. However, under warmer conditions, this export is projected to decrease and local retention of larvae is predicted to become proportionately more important. Similarly, the strong reliance of north‐eastern populations of *C. gunnari* on local retention in the present day is projected to further increase under climate change conditions. Present‐day simulations suggest extremely weak and intermittent direct export of *C. gunnari* from the Antarctic Peninsula to South Georgia and Shag Rocks; genetic connectivity is achieved by stepping stone transport via Elephant Island and South Orkney Islands. These island populations are also key for stepping stone transport of *N. rossii* from the Antarctic Peninsula to the South Sandwich Islands. Under warmer conditions, stepping stone connectivity via these routes is considerably reduced resulting in increased genetic isolation of South Georgia and Shag Rocks for *C. gunnari*, and of South Sandwich Islands for *N. rossii*. Identification of source and sink populations, key transport pathways, and understanding of the potential for climate change to restructure these systems, needs to be considered for fisheries and ecosystem management, and in the design of MPAs (Andrello, Mouillot, Somot, Thuiller, & Manel, 2015; Kough, Paris, & Butler, 2013). For example, protecting fish populations at South Georgia will have no impact on upstream populations at South Orkney Islands or around the Antarctic Peninsula. Similarly, it may be invalid to assume that populations at the Antarctic Peninsula will continue to seed fish populations at South Georgia as the climate warms. Thus, MPAs in scenarios of reduced connectivity may need to be more abundant and closer together in the future to achieve the same network effects expected today (Andrello et al., 2015).

### 4.3 Predicting response to climate change

Model simulations have highlighted the species‐dependence of the spatial scale over which changes in connectivity due to increasing temperatures impact the projected population genetic structure; whilst *N. rossii* populations remain broadly homogeneous except at the larger scale (> ~1,500 km), *C. gunnari* populations become more isolated, with the exception of the most proximate populations, such as those around the Antarctic Peninsula. These results suggest that the impact of climate change on the connectivity of Antarctic fish populations, and fragmented fish populations in general, cannot be assumed to be the same for all species. In particular, the species response likely depends on species‐specific early life‐history characteristics including planktonic duration and the temperature dependence of growth and mortality rates. In addition, the impacts of modified connectivity will likely be exacerbated by other biological, ecological and evolutionary factors such as larval growth and subsequent juvenile viability, and adult physiological and adaptive capabilities, including changes to reproductive output and the timing of spawning, with subsequent consequences for food availability (the match–mismatch hypothesis; Edwards & Richardson, 2004; Hoegh‐Guldberg & Bruno, 2010). Such biological controls may be particularly relevant for Antarctic species that have evolved in a stable cold environment (Clarke et al., 2007). Polar stenothermal species may have increased physiological sensitivity and reduced adaptive capability to tolerate warmer water. Icefish, in particular, may be unable to adapt to warmer temperatures as they possess no haemoglobin and have developed adaptations that allow them to thrive in the cold, oxygen‐rich waters of the Southern Ocean (Eastman, 1993). Hence, although *N. rossii* and *C. gunnari* have remarkably similar distributions, *C. gunnari* may be particularly sensitive to any rise in temperature due to their lack of haemoglobin (Beers & Sidell, 2011). However, for both species increases in temperature would be expected to have greatest impact on the growth, development and resilience of populations in the northern parts of their range where temperatures are already highest.

The existence of locally adapted populations in these two species has not yet been assessed. Under the climate change scenario, the projected reduced influx of Antarctic Peninsula genotypes to northerly populations (breakdown of connectivity), combined with an increase in self‐recruitment and accentuation of differences in local environments could, hypothetically in evolutionary time, allow the retention and proliferation of adaptations that may allow local populations to survive (Lenormand, 2002). Nevertheless, the potential for further development of such adaptations in range margin populations may already be limited (Hoffmann & Sgro, 2011), whilst our view of the role of gene flow on local adaptation is rapidly changing (Tigano & Friesen, 2016). Nonrandom gene flow combined with spatial heterogeneity and habitat matching can have positive effects on the rate of local adaptation (Edelaar & Bolnick, 2012; Jacob et al., 2017), and intermediate levels of gene flow maximize local adaptation in temporally variable environments (Blanquart, Kaltz, Nuismer, & Gandon, 2013). Overall, the breakdown in connectivity among populations of *C. gunnari* and *N. rossii* may thus further hinder the adaptive potential of isolated populations. To fully appreciate the potential impacts of climate change on range margin populations, we need improved understanding of the adaptive structuring of populations and the mechanisms underpinning such structuring.

Changing rates and patterns of ocean circulation are a key influence on the connectivity of marine populations, and hence, there is a clear need to understand how such phenomena may change in future as natural and anthropogenic climate change progress. Unfortunately, our ability to reliably predict future changes in ocean circulation is limited, not least because key processes that control circulation occur on spatial scales significantly smaller than the resolution of most IPCC‐class climate models (e.g., Meijers, 2014). For example, mesoscale eddies have horizontal scales of just a few tens of km or less in the Southern Ocean and are commonly represented in climate models in parameterized form (e.g., Gent & Mcwilliams, 1990). Despite these restrictions, fine‐scale model analyses and our understanding of the underlying ocean dynamics allows at least informed speculation concerning how patterns of circulation may change in future. On the large (circumpolar) scale, IPCC models generally agree that Southern Ocean winds will intensify and shift polewards in coming decades, in response to continued anthropogenic forcing (Meijers, 2014). The dynamics of the response of the ACC to changes in wind forcing are complex and nonlinear, but it is reasonably well established that extra energy imparted from winds will likely generate an increase in mesoscale eddy activity, with little or no long‐term change in overall ACC transport expected (Meredith & Hogg, 2006). Current understanding also suggests that wholesale southward shifts in the ACC current cores would be unlikely (Gille, 2014), although smaller scale changes to circulation patterns may occur. For example, Renner et al. (2012) studied the response of regional‐scale transport pathways in the vicinity of the Antarctic Peninsula under different wind forcing conditions, and found greater northward transport under stronger wind forcing, and changed pathways of mean circulation.

Such studies suggest that whilst the mean direction and strength of transport pathways over basin scales are likely to remain broadly similar to the present day under future climate change, over smaller scales, such as around the Antarctic Peninsula, changes are likely to occur. Increased eddy activity may also enhance the horizontal dispersion of upper ocean biota, potentially modifying the patterns of inter‐island connectivity. To progress this area of connectivity research, it will be necessary to utilize ocean models that reliably resolve the small scales known to be important for controlling circulation pathways and rates (mesoscale eddies, boundary currents, shelf‐edge circulation features etc.), whilst simultaneously being able to run for the decades over which climatic change (both anthropogenic and natural) is relevant. Such models are in development, but are only now beginning to become widely available. In addition, there is a lack of data on the response of Antarctic fish species to changes in temperature, in part due to the logistical complexities of sampling and undertaking experimental work in the Southern Ocean. More detailed representation of oceanographic processes, and species‐specific evolutionary and physiological capabilities, is essential for furthering our understanding of the response of fragmented fish populations to a changing climate and will be key for the evaluation of fisheries management options (Cheung et al., 2010).

### 4.4 Concluding remarks

At the global level, biodiversity is experiencing fundamental shifts in distribution patterns, with range‐restricted species, such as those from polar habitats, affected the most, predominantly by range contractions (Parmesan, 2006; Urban, Tewksbury, & Sheldon, 2012). Whilst interconnected locally adapted regional populations may be expected to modulate the severity of climate change effects by migrating, or through evolution of novel adaptations, the relative isolation of species restricted to the Southern Ocean makes range shifts and the emergence of adaptive novelty problematic: they have “nowhere left to go.” Indeed, marked adaptation is unlikely as these species are living near physiological limits (Hoffmann & Sgro, 2011), especially when exposed to predicted rates of climate change. It is well recognized that predicting the impacts of climate change on connectivity is complex, and depends upon such factors as taxon‐specific variance in response to shifts in temperature or localized impacts of island topography and alignment. Nevertheless, our results indicate that connectivity among populations will be markedly reduced by accelerating temperatures, likely leading to a potential loss of ecological resilience, increased localized extinctions and further range contractions. In such circumstances, it follows that those islands that presently export high volumes of larvae, and are predicted to continue to do so in future climate change scenarios, should be the focus of conservation measures that promote long‐term resilience of larval supply**.**

Recently, it has been shown that at the macroevolutionary scale, the Antarctic Peninsula and neighbouring islands have acted as the evolutionary source of notothenioid species diversity, and the repeated export of colonizers to nearshore Antarctic continental regions (Dornburg, Federman, Lamb, Jones, & Near, 2017). Here, we show that these source‐sink dynamics are mirrored at the microevolutionary scale in *C. gunnari* and *N. rossii*. Critically, as predicted by Dornburg et al. (2017), we demonstrate, using population genetics and biologically relevant model simulations, that increasing temperatures will likely lead to a restriction, or even complete breakdown, in connectivity, and increased isolation of source locations for the regional species pool (Ricklefs, 1987).

## DATA ARCHIVING STATEMENT

Model data are archived at the British Antarctic Survey and will be made available on request through contacting Dr Emma Young.

## APPENDIX A

### Relationship between egg duration and temperature

The dependence of egg duration on temperature was calculated using a theoretical relationship described by Gillooly, Charnov, West, Savage, and Brown (2002) and further discussed by Hirst and Lopez‐Urrutia (2006):

$$\frac{t}{m^{1/4}}=\frac{4}{\left[a\left(T_{0}\right)e^{\left(\tilde{E}/kT_{0}^{2}\right)\left(T_{c}/\left(1+T_{c}/T_{0}\right)\right)}\right]},$$

where *t* is time to hatch (days), *m* is mass at hatch (g), *T*_*c*_ is temperature (°C), *T*_0_ is 273K, *a*(*T*_0_) is a normalization factor for development, independent of body size and temperature, and variable across taxa, *Ẽ* is the average activation energy for metabolic reaction, and *k* is Boltzmann's constant. This may be rewritten

$$\ln\left(\frac{t}{m^{1/4}}\right)=\ln\left(\frac{4}{a(T_{0})}\right)-\frac{\tilde{E}}{kT_{0}^{2}}\;\left(\frac{T_{c}}{1+T_{c}/T_{0}}\right),$$

and by plotting $\ln\left(t/m^{1/4}\right)$ against $\left(T_{c}/\left(1+T_{c}/T_{0}\right)\right)$, solutions for the intercept, $\ln\left(4/aT_{0}\right)$ and gradient, $-\tilde{E}/kT_{0}$ , may be found (Hirst & Lopez‐Urrutia, 2006). However, as there are no data for Antarctic fish species, neither the intercept nor the gradient are known. Therefore, a value for the gradient term of −0.105 was assumed, based on data on marine fish egg development (Pauly & Pullin, 1988). Species‐specific values for the intercepts for *Notothenia rossii* and *Champsocephalus gunnari* could then be calculated as follows:

1. Approximate present‐day egg durations (*t*) for each species were obtained from published literature.
2. A present‐day mean temperature for the Scotia Sea of 1°C was assumed.
3. Values for egg mass were estimated from observed egg diameters and assumed egg densities of 1.01 g/ml for pelagic eggs (*N. rossii*) and 1.03 g/ml for benthic eggs (*C. gunnari*).

Once solutions for each species had been obtained, the change in egg duration (*t*) associated with an increase in temperature (*T*_*c*_) could be calculated (Table 1).
