# Supplementary material for: Stepping stones to isolation: Impacts of a changing climate on the connectivity of fragmented fish populations
Source: Evol Appl. 2018 Mar 14;11(6):978–94. doi: 10.1111/eva.12613 (PMC5999207; doi:10.1111/eva.12613)
Supplement: Supplementary file 1 [file EVA-11-978-s001.docx]

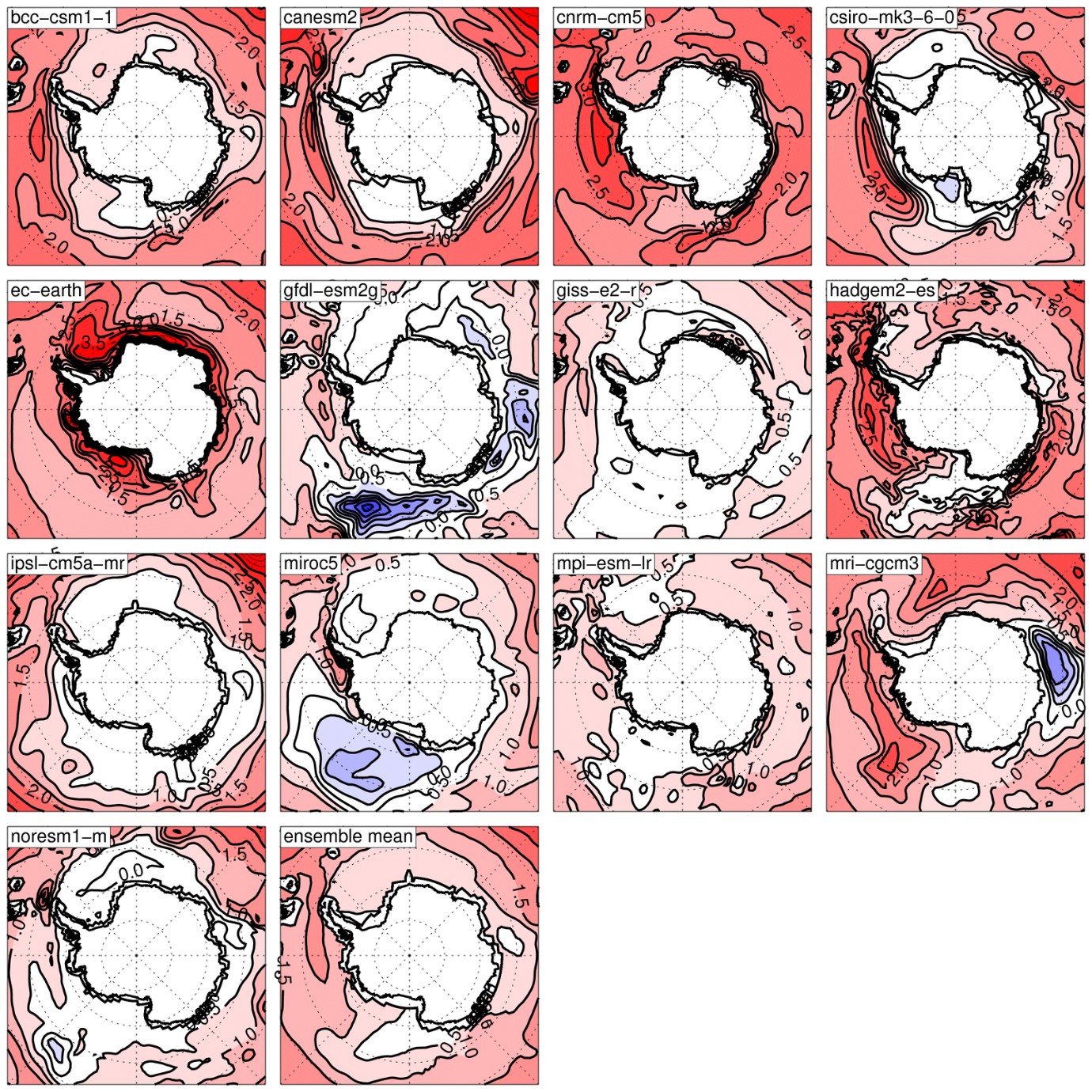


**Supplementary Figure 1**: Projected increase in annual mean sea surface temperatures (SST) for the Southern Ocean from a representative subset of climate models that were included in the Coupled Model Intercomparison Project Phase 5 (CMIP5). Temperature differences are between the end of the 21^st^ century (2070-2100) and the end of the 20^th^ century (1970-2000). The specific scenario used is one featuring strong greenhouse gas forcing (Representative Concentration Pathway 8.5). Further details of CMIP5, including the configurations of each climate model used here (named on panel insets), are available in Flato et al. (2013).

**Reference**

Flato, G., Marotzke, J., Abiodun, B., Braconnot, P., Chou, S. C., Collins, W., . . . Rummukainen, M. (2013). Evaluation of Climate Models. In T. F. Stocker, D. Qin, G.-K. Plattner, M. Tignor, S. K. Allen, J. Boschung, A. Nauels, Y. Xia, V. Bex, & P. M. Midgley (Eds.), *Climate Change 2013: The Physical Science Basis. Contribution of Working Group I to the Fifth Assessment Report of the Intergovernmental Panel on Climate Change* Cambridge, United Kingdom and New York, NY, USA: Cambridge University Press.
